# Assessment of the biofilm-forming ability on solid surfaces of periprosthetic infection-associated pathogens

**DOI:** 10.1038/s41598-022-22929-z

**Published:** 2022-11-04

**Authors:** Jung-Ah Cho, Yoo Jin Roh, Hye Rim Son, Hojung Choi, Jeong-Won Lee, Sung Jae Kim, Chang-Hun Lee

**Affiliations:** 1 School of Undergraduate Studies, College of Transdisciplinary Studies, Daegu Gyeongbuk Institute of Science and Technology, Daegu, 42988 Republic of Korea; 2 Department of Orthopedic Surgery, Dongtan Sacred Hospital, Hallym University, Hwasung, 18450 Republic of Korea; 3 Department of New Biology, Daegu Gyeongbuk Institute of Science and Technology, Daegu, 42988 Republic of Korea; 4 Department of Chemistry, Hanyang University, Seoul, 04762 Republic of Korea; 5 Department of Mechanical Engineering, Chosun University, Gwangju, 61452 Republic of Korea; 6 New Biology Research Center, Daegu Gyeongbuk Institute of Science and Technology, Daegu, 42988 Republic of Korea

**Keywords:** Bacteria, Biofilms

## Abstract

Biofilm formation is one of the leading causes of complications after surgery in clinical settings. In this study, we profiled the biofilm-forming ability of various periprosthetic infection-associated pathogens on medically relevant surfaces, polystyrene (PS) and titanium (Ti). We also explored how a specific environmental stressor, epigallocatechin gallate (EGCG), affected biofilm formation. First, Congo red tests revealed that all microorganisms formed biofilms within 72 h. Then, the amounts of biofilm formation on PS at 24, 48 and 72 h and also on a Ti plate for 72 h were determined. Some microbes preferred one surface over the other, whereas other microbes formed consistent levels of biofilm regardless of the surface material. *Staphylococcus lugdunenensis* was the most potent, while *Enterococcus faecalis* and *Staphylococcus aureus* were the weakest. Bacterial adhesion to hydrocarbon (BATH) tests indicated that the biofilm-forming abilities were not directly correlated with cell surface hydrophobicity (CSH). Finally, an external signal, EGCG, was applied to challenge the biofilm formation of each microorganism. EGCG regulated each microorganism’s ability differently, though the change was consistent across surfaces for most pathogens. This study can help a better understanding of a broad spectrum of periprosthetic infection-associated pathogens by relative comparison of their biofilm-forming abilities.

## Introduction

Biofilms are organized bacterial communities embedded in extracellular matrix composed of self-produced extracellular polymeric substances such as polysaccharides, proteins, lipids and DNA^1^. Various stimuli induce bacteria to form biofilms on a surface^2^ and behave differently from the planktonic growth mode^3^. Dangerous bacterial infections such as surgical site infections are attributed to these bacteria, which are protected from common antibiotics^4,5^. Bacteria especially form biofilms on medical devices like implants, causing catheter-associated urinary tract infections, peri-implant mucositis, and peri-implantitis^6–8^. A better understanding of biofilm formation can inspire strategies to prevent these infectious diseases.

Biofilms can be formed by not only bacteria that invade from the outside but also the commensal bacteria that become pathogenic^9^. Commensal bacteria protect the host under normal physiological conditions from colonization and invasion of pathogens by producing antimicrobials and competing for nutrients or adhesion sites^10^; this changes under pathological or immunologically-compromised conditions, where they may promote inflammatory diseases that damage the host^11^. *Staphylococcus aureus* and *Pseudomonas aeruginosa* are the two representative bacteria species identified as major causes of serious infectious diseases^12^. *Staphylococcus aureus* typically acts as a commensal of the human microbiota, which is found in normal skin flora, mucosa, and the reproductive tract^13–15^. However, this bacterium can cause various illnesses from mild infections to life-threatening diseases. Antibiotic-resistant strains like methicillin-resistant *S. aureus* are particularly problematic in clinics^16^. *Pseudomonas aeruginosa* is an opportunistic pathogen among immunocompromised individuals^17^; although it is not as virulent as *S. aureus*, *P. aeruginosa* is the most frequent colonizer of medical devices such as catheters ^18^. Coagulase-negative *staphylococci* such as *Staphylococcus lugdunensis* also cause many periprosthetic joint infections^19^. Other notable pathogenic bacteria associated with medical implants include *Streptococcus a*nd *Enterococcus* species^20^.

Various biological mechanisms regulate biofilm formation. One representative example is quorum sensing, which is the ability to regulate gene expression in response to cell population density^21^. The structural characteristics of microorganisms also determine biofilm formation^22^. Indeed, biofilm is the integrated result of a bacterial community that includes internal and external factors. However, each microbe within it may have a unique ability to form a biofilm by responding differently to the same environmental signal. Yet most studies about biofilms have focused on one specific or a few species^23–25^. We believed that a collective approach is needed to comprehensively understand the biofilm-related behaviors of a bacterial community. Through this study, we tested a broad spectrum of medically-relevant pathogens for their ability to form biofilms and attempted to confirm which microorganisms were robust in biofilm formation under conditions similar to actual clinical situations.

In this study, we studied the abilities of pathogens to form biofilms on polystyrene (PS) culture tubes and titanium (Ti) plates, of which surfaces are often provided in medical fields. PS is a plastic resin used for medical device applications including transparent packaging, diagnostic components, cell culture dishes, and housings for test kits^26^. Ti is widely used as a material for biomedical implants and surgical devices^27^. Each pathogen showed the unique ability to form biofilms on PS and Ti, and responded differently to the challenge with an external stimulus using epigallocatechin gallate (EGCG). Among the tested microbes, *S. lugdunensis* had the most potent ability to form biofilm on both surfaces. Understanding the abilities of these medically relevant pathogens to form biofilms in a clinical setting is a prerequisite to overcoming dangerous infections in clinical settings.

## Results

### Preliminary investigation for biofilm formation

**Figure 1:**
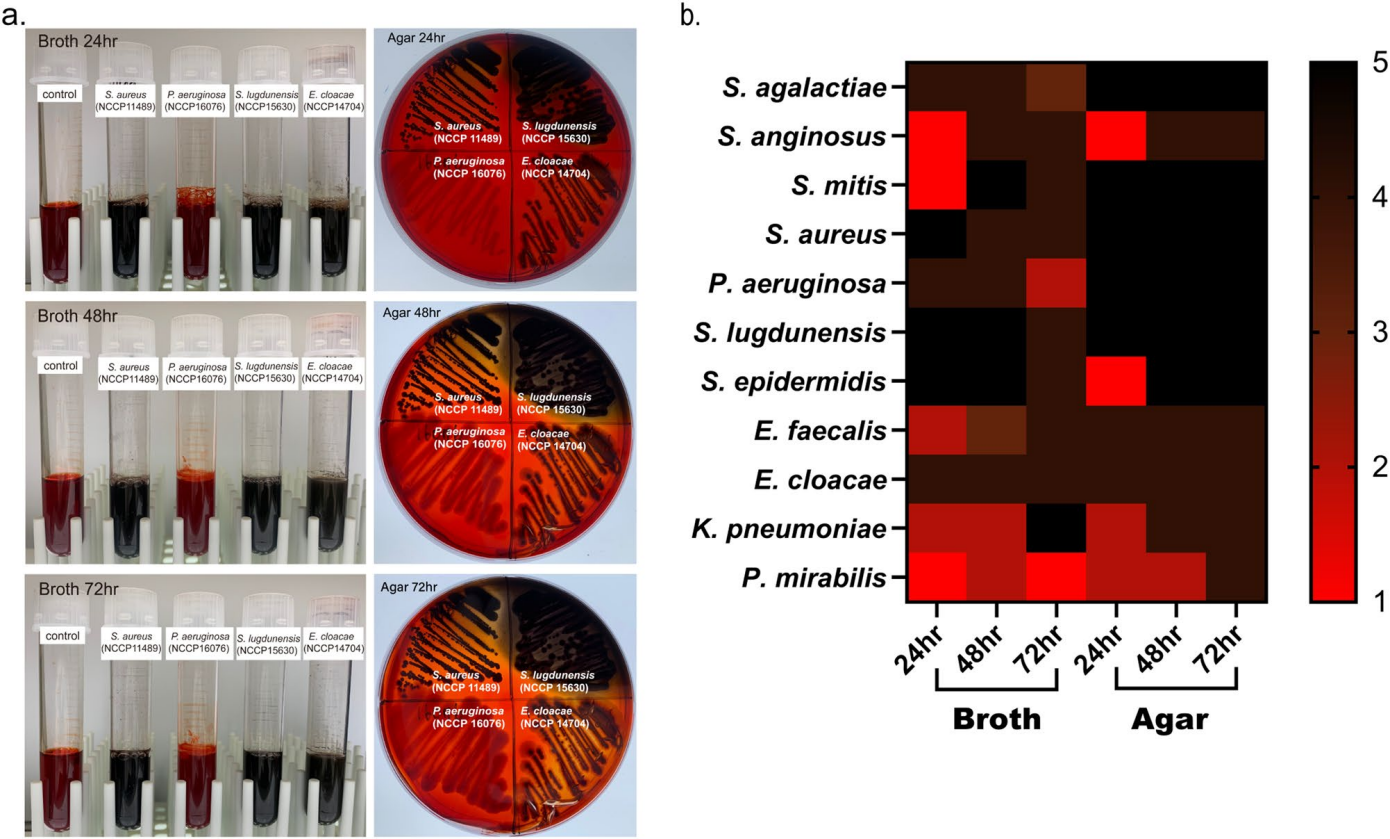
Congo red tests to screen biofilm production of microorganisms. (**a**). Representative images of Congo red tests with broth culture (left) and agar plates (right) at 24-, 48-, and 72-h time points. (**b**) Heatmap for Congo red test results of 11 microorganisms used throughout this study. Each number on the scale bar indicates the media color as follows: 1 = red, 2 = dark red, 3 = brown, 4 = dark brown, 5 = black.

We first assessed whether the microbes for this study could form biofilms because their capabilities were previously unknown (Supplementary table 1). Congo red test was performed to distinguish biofilm producers from non-producers (Fig. 1). In broth culture, the medium color of *S. agalactiae, S. aureus*, *P. aeruginosa* (NCCP 15783), *S. lugdunensis, S. epidermidis* *and E. cloacae* changed to brown or black after 24 h of incubation, while that of *S. anginosus*, *S. mitis*, *E. faecalis* and *K. pneumoniae* changed to brown or black after 48 h. *P. mirabilis* and the other *P. aeruginosa* (NCCP 16076) did not turn medium brown or black until 72 h. The non-biofilm-producing *P. aeruginosa* (NCCP 16076) was excluded from subsequent experiments. In agar culture, most of the test microbes including *S. agalactiae*, *S. mitis*, *S. aureus*, *P. aeruginosa*, *S. lugdunensis*, *E. faecalis* and *E. cloacae* changed to brown or black after 24 h of incubation, while *S. anginosus*, *S. epidermidis*, *K. pneumonia*, or *P. mirabilis* showed brown to black colonies after 48 or 72 h. Although all bacteria tested here eventually produced biofilm, they required different times to form biofilms (Fig. 1b).

We examined the growth curve of each microorganism to rule out the possibility that the differences in time required to form biofilms could be attributed to different bacterial growth rates (Supplementary Fig. 1). The time to reach the stationary phase was 6–12 h depending on the test microbe, and the extent of maximum growth ranged from 0.2 to 0.6 with an OD_600_ (Supplementary Table 2). Although *P. mirabilis* was shown to be the slowest to form biofilm in Fig. 1, the time to stationary phase (8 h) and OD_600_ at that time (0.44) was not lower than those of others. In other words, its growth rate and cellular amount were not the lowest among the test microbes. On the other hand, *S. aureus*, the fastest biofilm producer in Fig. 1, reached the stationary growth phase after 10–11 h, which was relatively late compared to other microorganisms. In addition, *S. epidermidis*, another fast biofilm producer in Fig. 1, was similar in growth rate and the extent to *P. mirabilis*, the slowest biofilm producer in Fig. 1. Therefore, it indicates that the timing and extent of biofilm formation shown in Fig. 1 resulted from each microbe’s innate biofilm-forming abilities rather than differences in the growth rates or cell numbers.

### Biofilm formation on polystyrene (PS) surface

**Figure 2:**
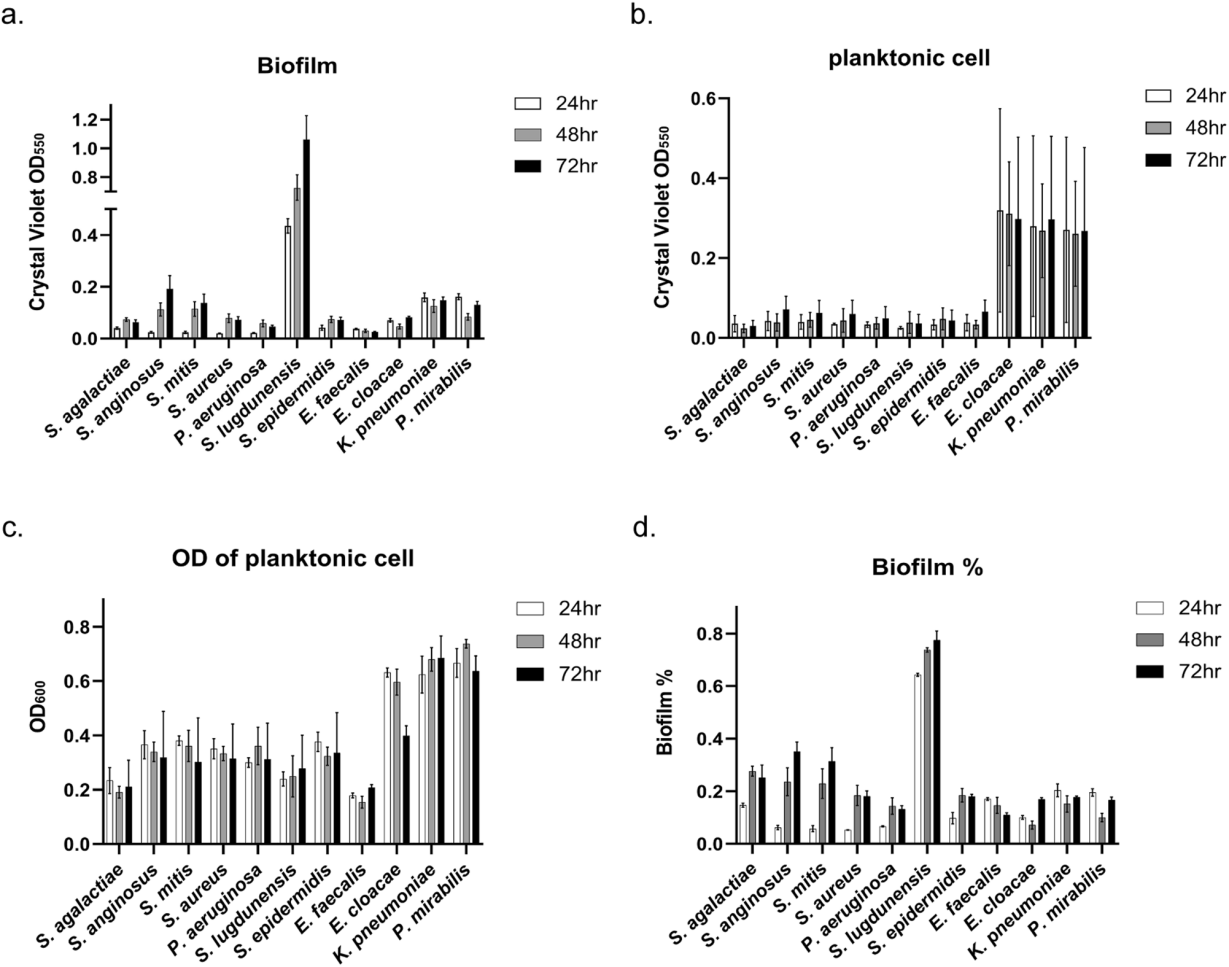
Biofilm formation on polystyrene (PS) surface. Each microorganism was cultured in PS tubes for 24, 48, or 72 h. Culture tubes and their supernatants were collected separately at each time point. (**a**) The culture tubes with biofilm were stained with crystal violet (CV), and their absorbance was measured at 550 nm to assess biofilm formation on the surface. (**b**) The pellets of planktonic cells in each supernatant were separately stained with CV. Their absorbances were measured at 550 nm to assess the amounts of planktonic cells in the tube. (**c**) The optical density (OD) of non-adherent planktonic cell-containing supernatants was measured at 600 nm. (**d**) The OD values obtained from the CV assay for biofilm formation were divided by the sum of OD values from the CV assay for biofilm formation on the surface and from planktonic cell-containing supernatant to calculate the ratio of biofilm formation.

PS is widely used in medical fields for a variety of medical applications, including diagnostic components, housings for test kits, and medical devices as well as used as a common surface for biofilm formation tests^23,26,28^. We cultured each microbe in a culture tube made of PS to examine their biofilm formation on this surface. The supernatant containing non-adherent planktonic cells and each culture tube containing biofilm were separately collected after 24, 48 and 72 h. Each supernatant’s optical density (OD) was measured at 600 nm to investigate each microorganism’s planktonic growth; each culture tube was subjected to a CV assay (measured at 550 nm) to quantify biofilm production, whose values are shown in Fig. 2a. Also, each planktonic cell in the supernatant was quantified through CV assay after being separated by centrifugation (Fig. 2b). The tested microorganisms showed different levels of biofilm formation on PS at 24-h culture. After that, some of them including *S. agalactiae* increased biofilm formation to 48 or 72 h, while others including *E. faecalis, E. cloacae*, *K. pneumoniae* and *P. mirabilis* tended to maintain their initial level for the entire experiment. The levels of biofilm formation on PS did not correlate with planktonic cell amount or growth (Fig. 2b,c). Interestingly, the planktonic cell level of *S. lugdunensis* was relatively low despite its high levels of biofilm, whereas *E. cloacae*, *K*. *pneumoniae* and *P. mirabilis* displayed relatively high levels of the planktonic cell compared to biofilm production. These results demonstrated that the degree of planktonic cell growth and the ability to form biofilm on PS are not directly connected. Indeed, biofilm production levels declined among microbes with high levels of planktonic cell growth (Fig. 2d). For comprehensive and intuitive understanding, we arbitrarily classified each microorganism into strong (over 0.2), intermediate (from 0.05 to 0.2), and weak (below 0.05) groups for the ability to form a biofilm on the PS surface according to the CV-stained values at the 72-h time point, (Table 1).

**Table 1:** Classification of each test microbe according to the degree of biofilm formation on PS and Ti.

Category	On PS	On Ti
(*P* < 0.0001)	Microorganism	Microorganism
Strong (0.2 ~)		*P. aeruginosa*
	*S.s lugdunensis*	*S. lugdunensis*
Intermediate (0.05 ~ 0.2)	*S. agalactiae*	*S. agalactiae*
	*S. anginosus*	*S. anginosus*
	*S. mitis*	*S. mitis*
	*S. aureus*	
	*S. epidermidis*	
	*E. cloacae*	*E. cloacae*
	*K. pneumoniae*	*K. pneumoniae*
	*P. mirabilis*	*P. mirabilis*
Weak (~ 0.05)	*P. aeruginosa*	*S. aureus*
		*S. epidermidis*
	*E. faecalis*	*E.s faecalis*

Microorganisms were classified on a subjective basis as strong, intermediate, or weak biofilm producers on PS if their absorbance values from CV stains were greater than 0.2, from 0.05 to 0.2, and less than 0.05, respectively. P-value < 0.0001 indicated statistical significance between groups (one-way ANOVA).

### Biofilm formation on titanium surface

**Figure 3:**
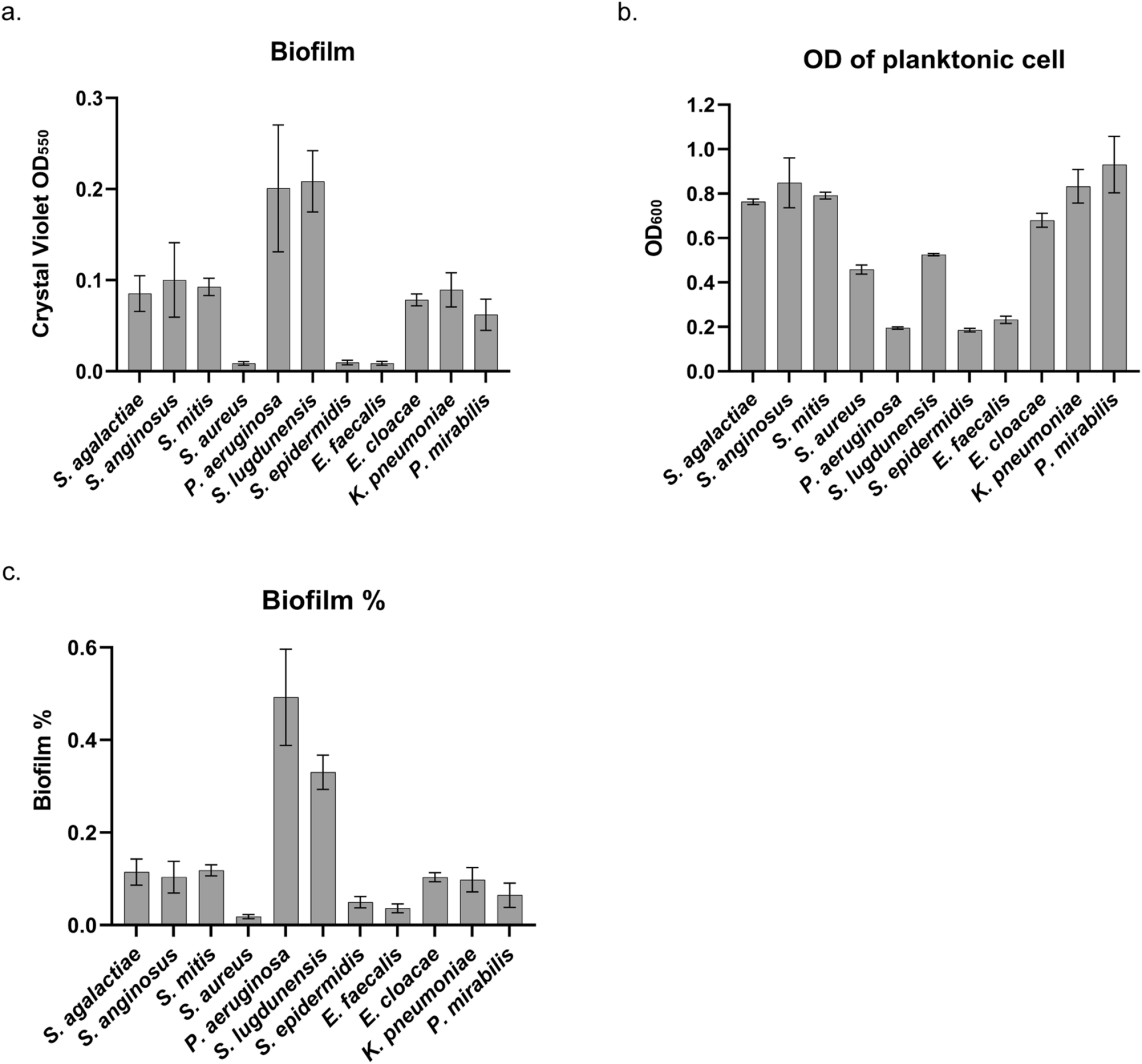
Biofilm formation on titanium (Ti). Each microorganism was cultured on a Ti plate for 72 h. Then the supernatants and Ti plates were collected separately. (**a**) The Ti plates were stained with CV, and their absorbance was quantified at 550 nm to detect biofilm formation on the surface. (**b**) The OD of non-adherent planktonic cell-containing supernatants was measured at 600 nm. (**c**) The OD values obtained from the CV assay for biofilm formation were divided by the sum of OD values from the CV assay for biofilm formation on the surface and from planktonic cell-containing supernatant to calculate the ratio of biofilm formation.

We then investigated the biofilm-forming abilities of the pathogens on titanium (Ti), an important and emerging biomaterial frequently used in prostheses^27^. After 72-h bacterial culture, Ti plates were separately collected to perform a CV assay, and the OD of each remaining culture supernatant was measured to quantify planktonic cell growth. From the results of the PS experiment, the OD of the supernatant and the CV assay results of the planktonic cells in each supernatant were proportional, so in the Ti experiment, the process of CV assay of planktonic cells was excluded. Like the PS results, each microorganism formed biofilm on the Ti surface to a different degree (Fig. 3a). *P. aeruginosa* and *S. lugdunensis* produced higher levels of biofilm on Ti, whereas *S. aureus, S. epidermidis* and *E. faecalis* showed weaker capabilities. Again, the amount of planktonic cell growth did not correlate with the level of biofilm formation in most of the test microorganisms (Fig. 3b). Notably, *P. aeruginosa* formed higher levels of biofilm but showed lower planktonic cell growth than others. When considering the degree of biofilm formation as a ratio to total bacteria, the ability to form biofilm was lower for microbes with high levels of planktonic cell growth (Fig. 3c). As previously done for PS results, each microorganism was relatively compared by classification into strong (over 0.2), intermediate (from 0.05 to 0.2), and weak (below 0.05) groups for capabilities to form biofilms on Ti according to the CV-stained values (Table 1).

### Analysis of the biofilm-forming activities

**Figure 4:**
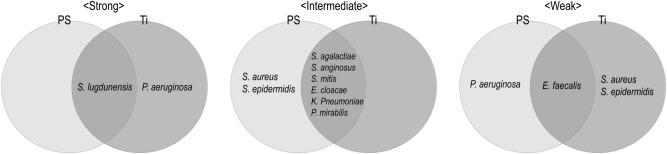
Comparison of biofilm-forming abilities on PS and Ti. The Venn diagram compares the biofilm-forming ability of each microorganism on PS or Ti according to its classification on each surface.

We compared the biofilm-forming abilities of the pathogens on PS or Ti surfaces and found both consistent patterns and lack thereof depending on the microorganism (Fig. 4). Among the test microbes, *S. lugdunensis* consistently and strongly formed biofilm on both PS and Ti. *S. agalactiae*, *S. anginosus*, *S. mitis, E. cloacae, K. pneumoniae* and *P. mirabilis* consistently formed intermediate levels of biofilm on both surfaces, while *E. faecalis* showed the weakest capabilities on both surfaces. The biofilm-forming abilities of the other microorganisms varied according to surface type. For example, *P. aeruginosa* formed high levels of biofilm on Ti but not on PS, and *S. aureus* showed the intermediate level of biofilm formation on PS but weak on Ti. It was clearly demonstrated that each microorganism regulated its inherent biofilm-forming ability according to a given surface.

### Evaluation of cell surface hydrophobicity

**Figure 5:**
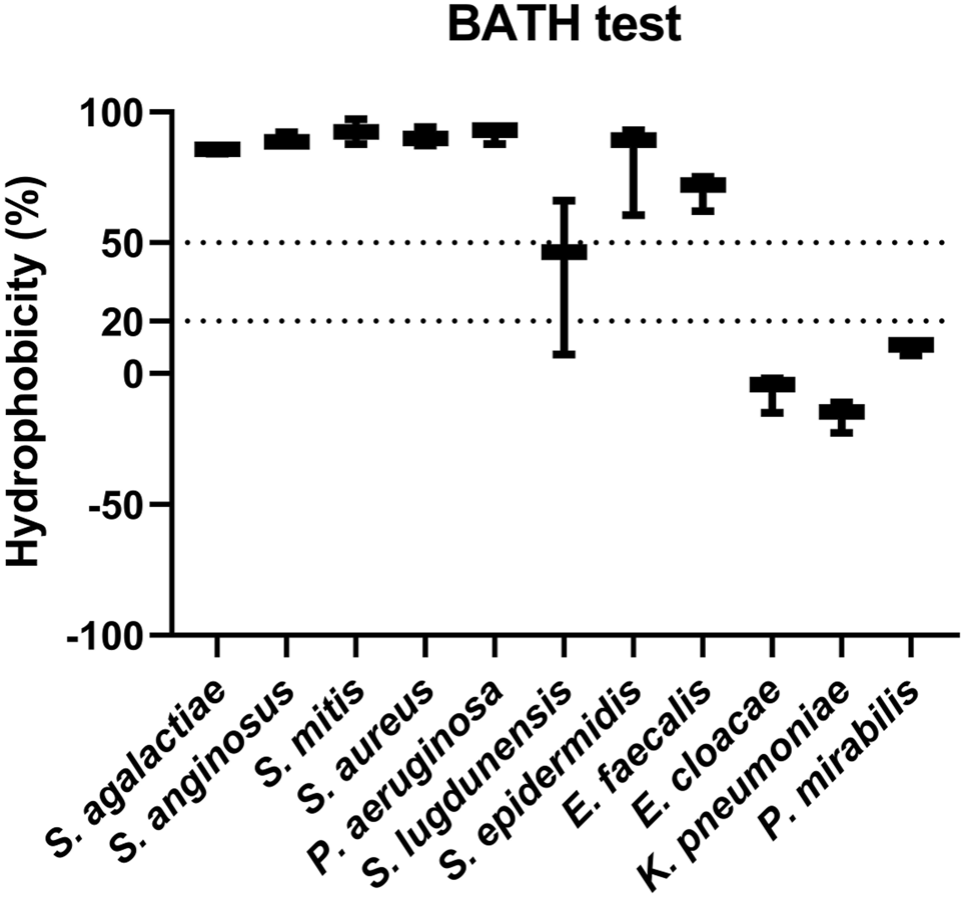
BATH tests for cell surface hydrophobicity. The bacterial adhesion to hydrocarbon (BATH) test was performed to measure each microorganism’s cell surface hydrophobicity (%). The experiments were repeated at least three times for each microorganism.

Cell surface hydrophobicity (CSH) was suggested to determine bacterial attachment on surfaces^29^, so we compared the CSH of each test microbe to its biofilm-forming abilities on the surfaces (Fig. 5). BATH tests showed a broad range of hydrophobicity among the microbes tested in this study. However, CSH did not correlate with the overall biofilm-forming ability of the microorganisms; the correlation analysis between CSH and CV values for biofilm showed that Pearson r and P value was -0.1882 and 0.5194 for PS surface, or -0.1693 and 0.6188 for Ti surface (Supplementary Fig. 2). Highly hydrophobic microorganisms, including *S. mitis*, and hydrophilic ones, including *K. pneumoniae*, formed intermediate levels of biofilm on both surfaces. *S. lugdunensis*, the most potent biofilm producer on both surfaces in this study, also had moderate hydrophobicity. This result indicated that CSH was not the primary determinant of the biofilm-forming ability of the tested pathogens on the two surfaces shown above.

### Effect of environmental factor on biofilm formation

**Figure 6:**
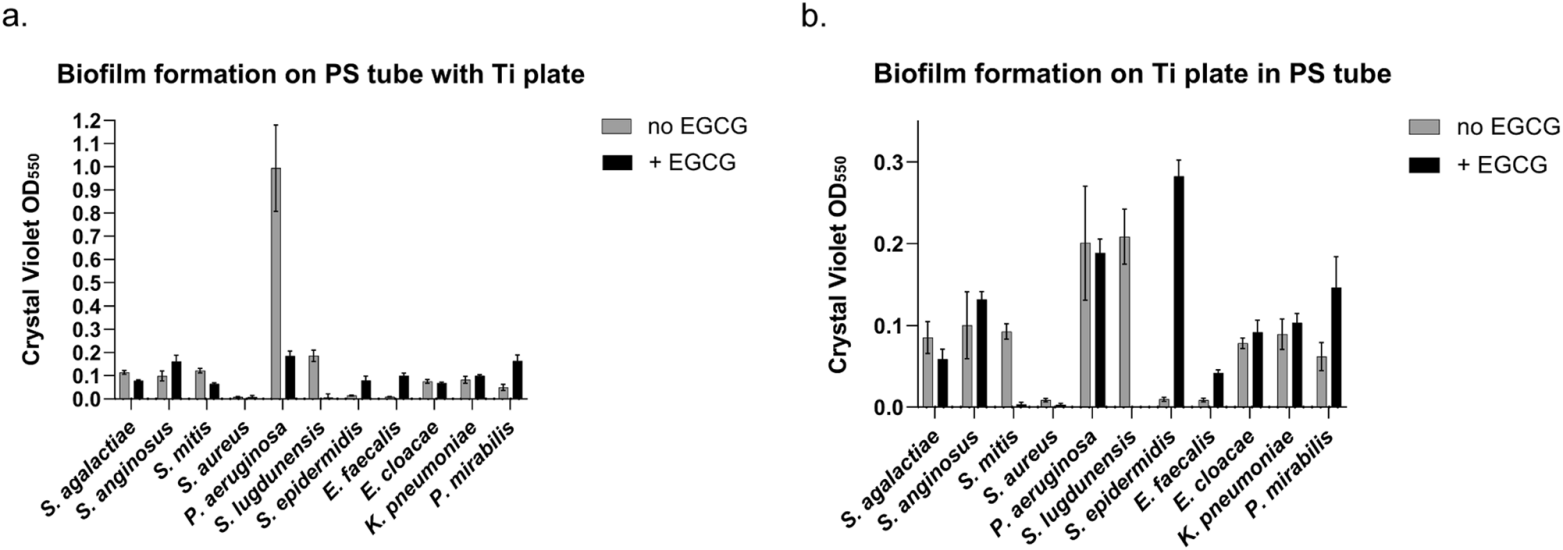
Epigallocatechin gallate (EGCG)-induced changes in biofilm formation on PS and Ti. Each microorganism was cultured to form biofilms on the surface of PS tube or Ti plate in the absence or presence of EGCG. (**a**) Biofilms on the PS tube surface were stained with CV, and their absorbance was measured at 550 nm. (**b**) Biofilms on the Ti plate surface were stained with CV, and their absorbance was measured at 550 nm.

Finally, we investigated how an environmental pressure can impact each pathogen’s ability to form biofilm on both surfaces. For this purpose, we chose EGCG as a survival inhibitor because it is a well-known anti-bacterial agent against a broad spectrum of microorganisms^30,31^. The microorganisms were cultured for 72 h in the presence of EGCG. Figure 6 shows the amounts of biofilm formation of each microorganism on the surfaces upon stimulation with EGCG. Among the tested pathogens, eight microorganisms increased or decreased biofilm production on PS after stimulation. On Ti, EGCG affected the biofilm production of nine microorganisms. EGCG consistently changed biofilm formation patterns across test surfaces for most microorganisms. *S. aureus*, *E. cloacae* and *K. pneumoniae* maintained biofilm production on both surfaces regardless of EGCG stimulation, while *P. aeruginosa* produced biofilm consistently only on Ti. Interestingly, EGCG hindered the biofilm formation of *S. lugdunensis*, the most potent biofilm producer among the test microorganisms, on both surfaces. Overall, the pathogens responded to EGCG independent of surface type. The results are summarized in Table 2.

**Table 2:** Grouping according to epigallocatechin gallate (EGCG)-induced changes in biofilm formation on PS or Ti.

Surface	PS	Ti
Decreased group	*S. agalactiae*	*S. Agalactiae*
	*S. mitis*	*S. Mitis*
	*S. lugdunensis*	*S. lugdunensis*
	*P. aeruginosa*	
Increased group	*S. anginosus*	*S. anginosus*
	*S. Epidermidis*	*S. Epidermidis*
	*E. Faecalis*	*E. Faecalis*
	*P. Mirabilis*	*P. mirabilis*
No change group	*S. aureus*	*S. aureus*
	*E. Cloacae*	*E. Cloacae*
	*K. Pneumoniae*	*K. pneumoniae*
		*P. aeruginosa*

Each group (Decreased, Increased, or No change) was assigned according to EGCG’s effect on biofilm formation.

## Discussion

Microbes survive by forming biofilms that threaten human health and lifestyle^32^. Biofilm can be formed on any surface through which water can flow, such as drains and boats^33^. Therefore, research on biofilm formation and prevention has especially garnered attention in fields related to aquatics and industrial water systems. Here, we characterized the biofilm-forming capabilities of life-threatening pathogens on surfaces commonly used in clinical environments, which can endanger patients^4,34^. This study is to relatively compare the intrinsic biofilm-forming capabilities of a broad range of clinically relevant pathogens. An interesting finding was that *S. aureus* and *P. aeruginosa* were not the most potent biofilm producers in this study despite their prevalence in biofilm-associated clinical situations^35^. Meanwhile, we found that among the microbes tested in this study, *S. lugdunensis* formed a relatively higher level of biofilm on both PS and Ti surfaces than others. Therefore, studies are needed to understand why *S. aureus* or *P. aeruginosa* is more often found as a major cause than *S. lugdunensis* in biofilm-related diseases.

When the biofilm-forming abilities were compared with planktonic cell growth in Figs. 2,3, we found no direct correlation between them because planktonic cell levels did not coincide with levels of biofilm formation. As revealed in the previously published studies by others that planktonic cell growth and biofilm formation involved different sets of molecules^36,37^, our finding also demonstrated that biofilm formation is a biological process independent and distinct from planktonic cell growth mode. Therefore, it is challenging to predict biofilm formation with the property of planktonic cell growth.

A single environmental signal could also affect the biofilm-forming ability of a single species. Epigallocatechin gallate (EGCG) is a phytochemical that is found in green tea extract, which has potent anti-bacterial and anti-biofilm activities against both gram-negative and gram-positive bacteria^38,39^. EGCG’s anti-microbial effects in various microbes include binding to and damaging the bacterial cell membrane via H_2_O_2_ generation^40^, disrupting bacterial membrane transporters^41^, inhibiting bacterial cell binding to host cells^39^, reducing bacterial H_2_S production^42^, and modulating bacterial enzymes including DNA gyrase^43^. EGCG also benefits human health through anti-inflammatory, anti-cancer, and antioxidant activities^44–46^. However, we found that EGCG can increase the biofilm formation of some microbes. The finding warns of the need to exercise caution in the usage of even a single chemical. We, therefore, suggest a pre-screening test for biofilm production of microbiota when a single agent is applied to a patient.

Although a single signal differentially regulated the biofilm formation of each microorganism, the changing pattern was consistent for both surfaces in most of the test microbes. This suggests that molecular expression rather than surface materials governs biofilm formation. This notion should be further tested on other surfaces. Interestingly, EGCG decreased the biofilm production of *S. lugdunensis*, the most potent biofilm producer among the test microorganisms, on both surfaces. *S. lugdunensis* is a coagulase-negative staphylococci that can cause severe infections, mostly in patients who use prosthetic devices^47,48^. EGCG also significantly downregulated the biofilm formation of *P. aeruginosa*, which was found to form a relatively higher level of biofilm on Ti. The application of EGCG is thus a potential anti-biofilm strategy for such cases as the surface coating of prosthetic implants. On the other hand, as mentioned above, the biofilm formation of some microbes, including *S. epidermidis*, especially on Ti, was upregulated by EGCG. The results obtained through EGCG stimulation present helpful guidance for the applicability of the chemical according to circumstances involving the biofilm of the test microbes.

Microbial CSH is reportedly critical for biofilm formation^29,49,50^; however, the CSH of the diverse microbes used here did not correlate with their biofilm-forming capabilities. This agrees with another study that found only a minor role for CSH in bacterial biofilm formation^51^. Yet external environmental factors can disrupt not only biofilm production but also CSH, which can lead to downstream molecular changes that determine biofilm formation. The interplay of these variables should be further characterized in future studies.

The composition of the bacterial community likely determines biofilm formation through a complicated network of biological signals. This warrants the need for precision medicine, as individuals with different microbiota may respond differently to an external signal. In this respect, this study has significance in providing a catalog with the biofilm-forming capabilities of various microorganisms on clinically relevant surfaces. Our work can help many future studies that will guide clinicians in preventing life-threatening conditions in clinical settings. In addition, this study can provide practical applications for future studies on bacterial biofilm. First, this study suggests the usage of *P. aeruginosa* when selecting a subject for biofilm-associated studies close to clinical models. Because of the clinical incidence, many biofilm-associated studies have used *S. aureus* as the sole test subject. However, this study suggests that the single use of *S. aureus* may be misleading with results. Even with a simple comparison in our studies, *P. aeruginosa* formed a more considerable amount of biofilm on a given surface when *S. aureus* did not show significant biofilm formation. Most importantly, this study clearly showed that biofilm formation could be different according to the material of the surface provided. Therefore, although PS has been widely used as a surface material for biofilm formation in previous studies, we suggest that a surface material for biofilm-associated studies should be appropriately selected according to the purposes of interest.

## Materials and methods

### Microorganisms

The species of microorganisms used in this study (Supplementary Table 1) are selected due to their frequency found in periprosthetic infections. The microbes were obtained from National Culture Collection for Pathogens (NCCP, Korea). Tryptic soy agar (TSA, Difco) or tryptic soy broth (TSB, Difco) was used to culture bacteria unless specified. Primary bacterial culture was prepared by inoculating one single colony on agar plates into broth media and incubating overnight at 37 °C with shaking at 120 rpm.

### Congo red test to distinguish biofilm producers from non-producers

The Congo red broth and agar assay were prepared as described in other studies^52,53^. Briefly, TSB or TSA medium was prepared with additional 3.6% sucrose (SIGMA S0389) and 0.08% Congo red dye (SIGMA C6277). A loopful inoculum of the bacterium from overnight broth culture was streaked onto a Congo red-containing TSA plate for the agar test. One colony of bacteria on a TSA plate was inoculated on the Congo red-containing TSB for the broth test. The colors of colonies or broth were observed after 24-, 48-, and 72-h incubations. Brown to black colors were considered positive for biofilm formation.

### Biofilm formation on surfaces

All experimental procedures or materials, devices and equipment were aseptically performed or maintained; a lack of cross-contamination was confirmed using empty plates. The primary culture was diluted with fresh broth media to achieve an optical density (OD) at 600 nm (OD_600_) value of 0.9 to 1.0 (DeNovix DS-C Spectrophotometer) for the biofilm formation test on PS. For this test, we used 14-mL round-bottom tubes (40114; SPL Life Sciences, Korea) made of PS which is commonly used in biofilm-forming experiments^22,25^. A 1-mL diluted bacterial suspension was dispensed in a 14-mL round-bottom tube that was sterilized by gamma irradiation, followed by incubation at 37 °C with shaking at 50 rpm^54^ for 24, 48 and 72 h. Then the non-adherent planktonic cells and the culture tubes were separately collected.

Rectangular TiAl6V4 plates (Grade 23: 90% Titanium, 60% Aluminum, 4% Vanadium, 20 width × 16 height mm; Jeil Medical Corporation, Korea) were prepared for the biofilm formation test on Ti by submerging them in acetone and rinsing with ultrapure water, followed by autoclaving at 121 °C for 20 min with a pressure of 15 psi. After drying in a dry oven at 60 °C, the autoclaved rectangular Ti plate was placed on the bottom of a 50-mL conical tube. After diluting the primary culture 100-fold (OD_600_ = approximately 0.01) with fresh broth, a 5-mL aliquot of the diluted bacterial suspension was added to the Ti-containing tubes; the Ti plate was halfway submerged to form a biofilm at the air–liquid interface in the middle. The caps were tightly closed during incubation at 37 °C with shaking at 50 rpm^55^ for three days. Finally, the Ti plates were separately retrieved from the non-adherent planktonic cells and culture tubes.

### Crystal violet assay

Biofilm formation was quantified by staining with crystal violet dye (CV; SIGMA V5265; Sigma-Aldrich, USA) as previously described^56^. After washing with autoclaved ultrapure water three times, the planktonic cell pellets, plates, or tubes were placed in 0.1% CV solution for 10–15 min, followed by another three washes with autoclaved ultrapure water. The CV-stained biofilms and planktonic cell pellets were air-dried and then dissolved in 30% acetic acid solution (DUCKSAN 414 extra pure grade, Korea) for 15–20 min. The solubilized fractions were transferred in triplicate (100 μl each) to 96-well plates, and their absorbances (OD_550_) were assessed at 550 nm^57,58^ using a UV–Visible spectrophotometer (TECAN SPARK™ 10 M Multimode Plate Reader with SPARKCONTROL). Data were analyzed from repeated experiments (N = 2 ~ 3) including *n* = 9 for PS and *n* = 4 for Ti, respectively.

### BATH (Bacterial Adhesion to Hydrocarbon) test for determining cell surface hydrophobicity (CSH)

Microbes were incubated in 4 mL of TSB for 24 h at 37 °C and 120 rpm. The overnight bacterial culture was washed with phosphate-buffered saline (PBS; SIGMA D8537) twice as follows: microbes were pelleted by centrifugation at 4000 rpm for 5 min, and then the supernatant was removed before resuspending it in PBS. After measuring the absorbance at 600 nm [A] in triplicate, the bacterial culture was added with 1 mL of decane (SIGMA-ALDRICH D901) and mixed thoroughly by vortexing for 1 min, followed by standing for 15 min at RT. The upper decane-containing phase was removed, and the absorbance of the remaining clear phase was measured at 600 nm [B] in triplicate. Results were analyzed using Eq. (1). Microbes with hydrophobicity below 20%, between 20 and 50%, and above 50% were defined as hydrophilic, moderately hydrophobic, and highly hydrophobic, respectively.

1 $$Hydrophobicity \left(\%\right)= \frac{A-B}{A}\times 100.$$

### Statistical analysis

Each microorganism was divided by subjective criteria into strong, intermediate, or weak group based on the values obtained from the CV assay to intuitively understand each microbe’s biofilm-forming ability through relative comparison. Unpaired *t* test and ordinary One-way ANOVA were performed using GraphPad Prism 9 (GraphPad Software Inc., USA) to assess significant differences between groups. The level of significance between each group was P < 0.0001.

### Tests for effects of an external signal

EGCG (SIGMA-ALDRICH PHR1333) was dissolved in sterilized distilled water to make a 10 mM stock. Then the stock solution was filtered twice through a 0.45 μm syringe filter and stored at 4 °C until use. The proper amount of stock solution was added to the culture media to obtain a final concentration of 1 mM, which was determined in our unpublished preliminary experiments.

### Measurement of bacterial growth curve

One colony of each microorganism on the agar stock plate was inoculated into TSB media (5 ml) and incubated overnight at 37 °C. The overnight bacteria culture (250 ul) was then diluted at 1:100 (OD_600_ = approximately 0.01) into new TSB media (25 ml), and optical density (OD) was measured at 600 nm (time 0) using a spectrophotometer, followed by incubation at 37 °C. Aliquots (1 ml) of the bacteria culture suspension were taken and monitored at one-hour intervals for the growth rate by measuring OD at 600 nm.

## Supplementary Information

Supplementary Information.

## Data Availability

All the datasets generated or analyzed during this study are available from the corresponding authors on reasonable request.
