# Supplementary material for: Assessment of the biofilm-forming ability on solid surfaces of periprosthetic infection-associated pathogens
Source: Sci Rep. 2022 Nov 4;12:18669. doi: 10.1038/s41598-022-22929-z (PMC9636376; doi:10.1038/s41598-022-22929-z)
Supplement: Supplementary file 1 — Supplementary Information. [file 41598_2022_22929_MOESM1_ESM.pdf]

Supplementary information

Supplementary table 1. Microorganisms used in this study.

Bacteria strain	Designation	Isolated from	Remarks
Staphylococcus aureus	NCCP 11489	Blood	Methicillin-resistant
Pseudomonas aeruginosa	NCCP 15783	Sputum	
Pseudomonas aeruginosa	NCCP 16076	Sputum	Only used in the preliminary Congo red test
Staphylococcus lugdunensis	NCCP 15630	Pus	
Staphylococcus epidermidis	NCCP 14768	Blood	
Streptococcus agalactiae	NCCP 14728	Blood	
Streptococcus anginosus	NCCP 14730	Blood	
Streptococcus mitis	NCCP 14733	Blood	
Enterococcus cloacae	NCCP 14704	Blood	
Enterococcus faecalis	NCCP 15611	Blood	
Klebsiella pneumoniae	NCCP 14713	Blood	
Proteus mirabilis	NCCP 14719	Blood	

Link for more information; https://nccp.kdca.go.kr/eng/main.do#

Measurement of the bacterial growth curve.

One colony of each microorganism on the agar stock plate was inoculated into TSB media (5 ml) and incubated overnight at 37°C. The overnight bacteria culture (250 ul) was then diluted at 1:100 (OD = approximately 0.01) into new TSB media (25 ml). Optical density (OD) was measured at 600 nm (time 0) using a spectrophotometer, followed by incubation at 37°C. Aliquots (1 ml) of the bacteria culture suspension were taken and monitored at one-hour intervals for the growth rate by measuring OD at 600 nm as done at time 0.

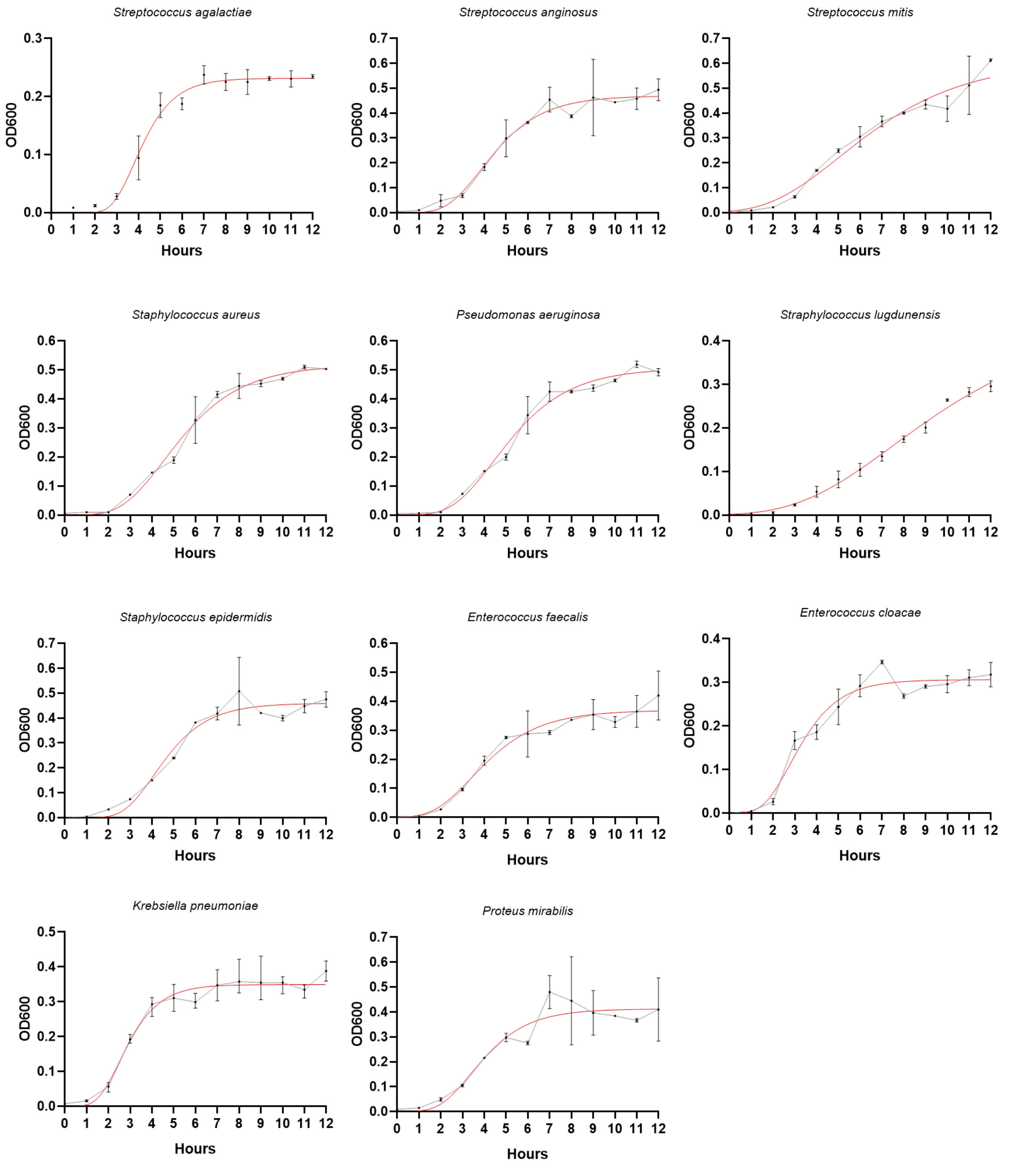

Supplementary figure 1. The growth curves of each microorganism. Each bacterial growth rate was monitored for twelve hours by measuring their optical density at OD 600nm (OD_{600}) at one-hour intervals. Graphs were generated and analyzed by using Prism8 software. Each black dot points mean OD values + SD errors of 2-3 independent samples measured at 600 nm at the indicated time point, and the solid red line represents the growth curve based on the OD_{600} values.

Supplementary table 2. The time to reach the stationary phase and corresponding absorbance value for each microorganism.

Strain	ID	Time to stationary phase (hr)	OD 600 (mean)
Streptococcus agalactiae	NCCP 14728	8	0.225
Streptococcus anginosus	NCCP 14730	7	0.454
Streptococcus mitis	NCCP 14733	12	0.612
Staphylococcus aureus	NCCP 11489	11	0.5095
Pseudomonas aeruginosa	NCCP 15783	12	0.492
Staphylococcus lugdunensis	NCCP 15630	12	0.295
Staphylococcus epidermidis	NCCP 14768	8	0.5075
Enterococcus faecalis	NCCP 15611	10	0.3285
Enterococcus cloacae	NCCP 14704	6	0.292
Krebsiella pneumoniae	NCCP 14713	7	0.2722
Proteus mirabilis	NCCP 14719	7	0.48

The bacterial growth rate was monitored by measuring the optical density at 600 nm (OD_{600}) at one-hour intervals. The time point (hr) at which the stationary phase was achieved and the corresponding absorbance mean value were recorded.

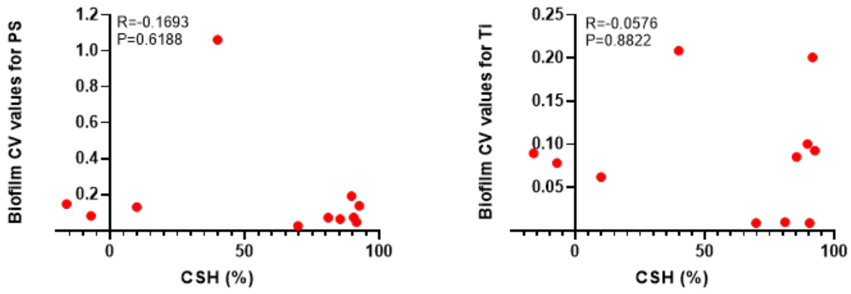

Supplementary figure 2. Correlation analysis between CSH and CV value for biofilm on surfaces. Each dot indicates the CSH and CV value of one microorganism. CSH (x-axis) and CV-stained values for biofilm formed on PS or Ti surface (y-axis) were subjected to a correlation analysis using Prism8 software.
